# Lymphatic transport of exosomes as a rapid route of information dissemination to the lymph node

**DOI:** 10.1038/srep24436

**Published:** 2016-04-18

**Authors:** Swetha Srinivasan, Fredrik O. Vannberg, J. Brandon Dixon

**Affiliations:** 1 School of Biology, Georgia Institute of Technology, Atlanta, GA, USA; 2 Parker H. Petit Institute for Bioengineering and Bioscience Georgia Institute of Technology, Atlanta, GA, USA; 3 George W. Woodruff School of Mechanical Engineering, Georgia Institute of Technology, Atlanta, GA, USA

## Abstract

It is well documented that cells secrete exosomes, which can transfer biomolecules that impact recipient cells’ functionality in a variety of physiologic and disease processes. The role of lymphatic drainage and transport of exosomes is as yet unknown, although the lymphatics play critical roles in immunity and exosomes are in the ideal size-range for lymphatic transport. Through *in vivo* near-infrared (NIR) imaging we have shown that exosomes are rapidly transported within minutes from the periphery to the lymph node by lymphatics. Using an *in vitro* model of lymphatic uptake, we have shown that lymphatic endothelial cells actively enhanced lymphatic uptake and transport of exosomes to the luminal side of the vessel. Furthermore, we have demonstrated a differential distribution of exosomes in the draining lymph nodes that is dependent on the lymphatic flow. Lastly, through endpoint analysis of cellular distribution of exosomes in the node, we identified macrophages and B-cells as key players in exosome uptake. Together these results suggest that exosome transfer by lymphatic flow from the periphery to the lymph node could provide a mechanism for rapid exchange of infection-specific information that precedes the arrival of migrating cells, thus priming the node for a more effective immune response.

Multicellular organisms rely on cell-cell communication for information exchange in order to promote survival and appropriate development and functioning of tissues. This communication occurs either through direct physical contact via nanotubes^1^, secreted chemical signals like cytokine, chemokines, or small molecule mediators (proteins, nucleic acids), or exchanging information via exosomes^2^. Exosomes provide the ability to transmit messages between cells at a distance and their roles in long distance communication have been well established^3^. The discovery of functional, transportable mRNA and miRNA within exosomes further increases the complexity of cell-to-cell communication. They can fuse with the recipient cells and deliver their contents into the cytoplasm of the recipient cell and perturb the recipient cell, especially since miRNA can mediate RNA interference^4^. They can also bear combinations of ligands to engage several cellular receptors at once modulating changes in the recipient cell.

Exosomes are credited with several roles in modulating immune response *in vivo* :a) dendritic cell derived exosomes carry antigens and present them to T-cells^5^, b) exosomes from macrophages infected with intracellular pathogens induce a pro inflammatory response in uninfected macrophages thereby activating the immune response^6^ and c) tumor derived exosomes carry a variety of immunosuppressive molecules to suppress the immune response to the tumor by decreasing proliferation of various immune subsets like natural killer cells, regulatory T-cells or myeloid cells^7^.

Lymphatic flow is an important component of the circulation as it serves to return interstitial fluid from tissue back to the circulation via the lymph nodes and thoracic duct^8^. Lymphatic drainage from tissue results in transport of antigens, immune cells and large macromolecules from the periphery to the lymph nodes where innate and adaptive immune responses are elicited. Thus, each lymph node obtains region specific antigenic information through the lymphatic capillaries that drain the periphery, allowing antigen presenting cells (APCs) to initiate an immune response^9^. Interestingly, the intrinsic physical barriers created by the interstitium and vascular exclusion of large proteins, create an “optimal” size range for lymphatic transport of 5–100 nm which is primarily the size range of exosomes. Particles smaller than this are easily taken up in the blood capillaries and larger particles typically become trapped in the extracellular matrix^10^,^11^, although more recent evidence suggests that particles as large as 1 micron could be taken up by lymphatics^12^.

It is likely that one of the primary advantages of exosome size is that they are small enough to convect through the interstitial matrix with interstitial flow, yet large enough to partition their uptake into the lymphatic circulation, thus making them an ideal vehicle through which a peripheral cell can rapidly signal and transport information to the lymph node. Interestingly, melanoma-derived exosomes were shown to prime the sentinel lymph node for tumor metastasis by initiating a proangiogenic program and remodeling the tissue matrix^13^ and CD169^+^ cells were identified as the target cells for B-cell derived exosomes in the lymph nodes and spleen^14^, implicating the lymphatic system in playing an important role in exosome transport from the periphery to lymphoid organs and the nodes. However, experiments involving exosome signaling in the node are typically conducted over the course of hours or days and thus it is unclear how rapidly exosome trafficking and uptake into cells in the node can occur, a process in which speed should be of particular importance if exosomes are utilized to enhance innate immunity.

Near-infrared imaging is an emerging technology and has been used to non-invasively image and quantify functional lymphatic transport^15^ and perform sentinel lymph node mapping^16^ as it offers maximum tissue penetration with minimal autofluorescence^17^. Exosomes on the other hand have been imaged by either covalently labeling with a fluorophore^18^ or with a variety of lipid dyes such as DiL or DiO^13^ in the visible range, which allows for trafficking of exosomes in cell cultures or endpoint *in-vivo* biodistribution studies of exosomes, but suffers from depth penetration limitations making them ill-suited for *in vivo* imaging. We have successfully labeled exosomes with a near-infrared dye which enables us to monitor exosome trafficking *in vivo* using near-infrared imaging. Thus, we can establish and quantify the kinetics of lymphatic transport of exosomes from the peripheral tissue to the lymph node, which is particularly important in the context of innate immunity where rapid antigen transport can be crucial to the establishment of host immunity and limiting pathogen spread^19^. Characterizing exosome trafficking through the lymphatics and the resulting cellular uptake in the lymph node provides several key insights into both the role of lymphatic drainage as well as paracrine effects of exosomes in the context of immunity.

## Results

### Characterization of exosomes and beads

**Figure 1:**
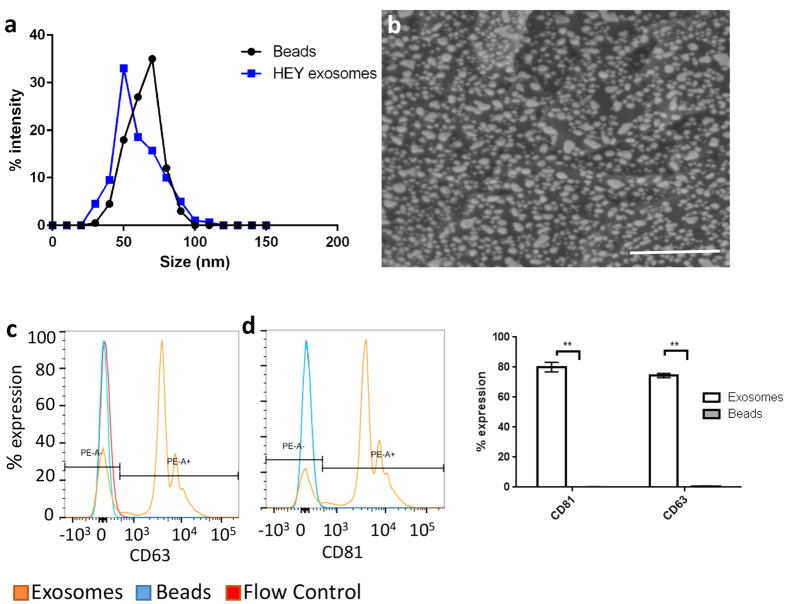
Characterization of exosomes and beads. (**a**) Size distribution of HEY exosomes as compared to that of beads. (**b**) Scanning electron micrograph of exosomes. Scale bar = 500 nm. (**c**) Expression of CD63 and (**d**) CD81 on exosomes and beads. (**e**) Quantitation of CD63 and CD81 on exosomes and beads by flow cytometry (p-value < 0.01).

Exosomes from the HEY cell line were isolated and characterized along with size and density matched polystyrene beads using dynamic light scattering, scanning electron microscopy and for surface marker expression using flow cytometry. The average size of HEY exosomes was 78.82 ± 19.17SD nm as compared to the beads which had an average size of 67.34 ± 13.7SD nm (Fig. 1a). Exosomes had a spherical shape with a diameter of ~60–75 nm as seen from scanning electron microscopy which agreed with previous reports of exosome shape and size reported in literature^20^ (Fig. 1b). The classical tetraspanin surface markers CD63 and CD81, which are known to be enriched on exosomal membranes^4^, had an ~80% expression level on HEY exosomes [Fig. 1c–e]. Thus, the HEY exosomes used in this study conformed to known exosomal size ranges, expressed the classical tetraspanin markers and were spherical in shape as previously reported.

### Exosomes are transported rapidly and selectively through the lymphatic endothelium *in vitro*

**Figure 2:**
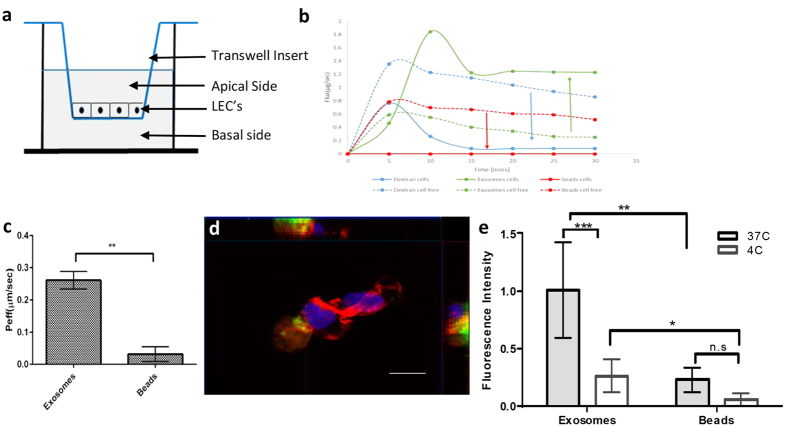
Exosomes transported rapidly and selectively through the lymphatic endothelium *in vitro.* (**a**) Schematic of transport experiment, (**b**) Transport of exosomes across the lymphatic endothelium occurs rapidly (t = 5–30 mins) and is enhanced in the presence of cells, (**c**) Exosomes are selectively transported into the lymphatic endothelium (versus beads), (**d**) Orthogonal view of LEC’s (nuclei stained with DAPI, actin stained red) with PKH67 exosomes and at 37 °C. Scale bar, 5 μm and (**e**) quantitation of exosome and beads in cells by fluorescence intensity at 37 °C and 4 °C.

To test the hypothesis that transport of exosomes across the lymphatic endothelium is higher than size and density matched beads, the effective permeability of cells (*P*_*eff_cell*_) to the fluorescently labeled exosomes and beads in the basal to apical direction was measured using a transwell system as described previously^21^ (Fig. 2a). Exosomes, beads and dextran were freely transported across the membrane in the absence of cells (Fig. 2b, dotted lines) and neither exosomes nor beads stuck to the membrane (Supp. Fig. 1d). Additionally, the size ranges of the exosomes collected on the apical side were similar to that on the basal side, further confirming exosome trafficking from the basal to apical sides of the LECs (Supp. Fig. 1e). To understand the kinetics of exosome transport by LECs, transport was assessed every 5 min. The transport of beads was below the detection limit at 30 min and therefore is represented as a solid line at the zero mark indicating no transport. Flux was calculated both in the presence and absence of cells to determine the extent that LECs enhanced or alternatively provided a barrier to selective transport. Dextran, being extremely small (3kDa) freely diffused through the Transwell membrane in the absence of cells, but transport was slightly reduced in the presence of LECs and rapidly reached equilibrium at about 15 min. Exosomes were rapidly detected across the lymphatic endothelium at 5 min and transport in the presence of cells was much higher than in the absence of cells (~2 fold) with transport reaching equilibrium at ~20 min (Fig. 2b, solid lines). In order to quantify this difference, the effective permeability of cells was calculated after incubation with exosomes and beads for 75 mins so transport could attain equilibrium at 37 °C and 4 °C. Exosomes were transported across the lymphatic endothelium ~10 times more as compared to the fluorescent size matched beads (p-value <0.01) at 37 °C (Fig. 2c). When the cells were fixed and examined using confocal microscopy, exosomes were seen within cells at 37 °C (Fig. 2d) whereas beads were not (Supp. Fig. 1b). However, exosome uptake was greatly reduced at 4 °C (Supp. Fig. 1a). The fluorescence in the images that corresponded to exosomes and beads was quantified at 37 °C and 4 °C which showed that exosome transport was reduced by ~80% at 4 °C (Fig. 2e, p-value <0.001). Collectively this data suggests that the lymphatics actively transported exosomes *in vitro*.

### Exosomes are rapidly transported into lymphatics *in vivo*

A tissue phantom was utilized to test the sensitivity of the NIR imaging system for detecting dual labeled exosomes. Exosomes were detected at a signal-to-noise ratio >4 at depths of 1–6 mm (Supp. Fig. 2b). We tested the limit of detection of exosomes within the vessels by running several dilutions of the exosome solution through the tissue phantom and were able to detect 0.1 μg of exosomes which is 1% of the injected dose of 10 ug (Supp. Fig. 2c). Lastly the lymph node phantom was able to detect an exosome concentration of 0.01 μg/μl exosomes, or 1% of the injected dose of 1 μg/μl (Supp. Fig. 2d).

**Figure 3:**
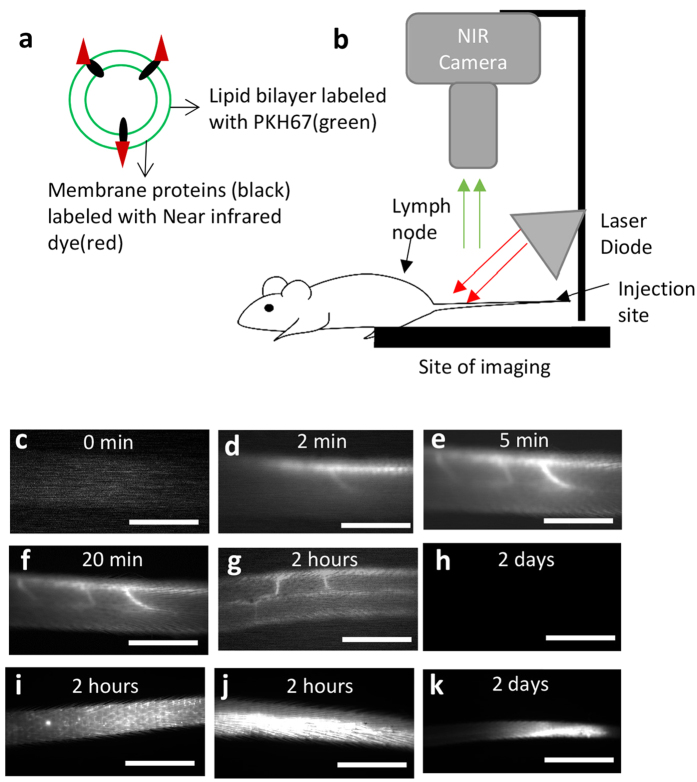
Exosomes are transported rapidly through the lymphatic endothelium *in vivo*. (**a**) Dual labeling of exosomes, (**b**) injection and visualization scheme in mice. Exosomes are detected in the lymphatics rapidly (**c**) vessel at 0 mins, (**d**) vessel at 2 mins, (**e**) vessel at 5 mins, (**f**) vessel at 20 mins (**g**) vessel at 2 hours, (**h**) vessel at 2 days, (**i**) lymphatic capillaries seen close to the injection site at 2 hours, (**j**) injection site at 2 hours, and (**k**) injection site at 2 days. Scale bar; 5 mm.

To track the movement of exosomes real time *in vivo*, a near-infrared fluorophore was conjugated to the N-terminal of exosomal membrane proteins. A second fluorophore was added in the lipid bilayer of the exosomes to enable *ex-vivo*, multi scale analysis of cellular exosome uptake and transport (Fig. 3a). Mice were injected intradermally with a 10 µg bolus of dual labeled exosomes in 10 µL of PBS. The near-infrared excitation source and the field of view of the CCD emission detector were centered on the mouse tail 10 cm downstream (toward the base of the tail) from the injection site at the tip of tail (Fig. 3b). This location ensured that only the downstream collecting lymphatics would be visualized so as to maximize detection sensitivity and avoid image saturation from the injection site (Fig. 3i).

Exosomes were seen in the lymphatic collecting vessels within 2 min of injecting the bolus in the tip of the tail 10cm downstream of the injection site (Supp. Video 1). Exosomes were detected first in the dominant vessel draining the tail, and then in the non-dominant vessel about 2.5 min later. Both vessels reached a steady state value of fluorescence by 20 min after injection (Fig. 3c–f). This result agreed with previous findings that reported lymphatic transport in rodent tails and mouse hind limbs where the two collecting vessels had varying functional capacity as measured by NIR imaging^22^,^23^. The collecting vessels maintained these steady state values for up to 6 hours post injection however there was no detectable signal that remained in the vessel 24 hours after injection. Representative images of the collecting vessels are shown at the 2 hour and 2 day time points, the end points of our study (Fig. 3g,h respectively). The injection site continued to retain a fraction of the injected exosomes at 2 hours and 2 days (Fig. 3j,k respectively).

### Characterization of exosomes transport *in vivo*

**Figure 4:**
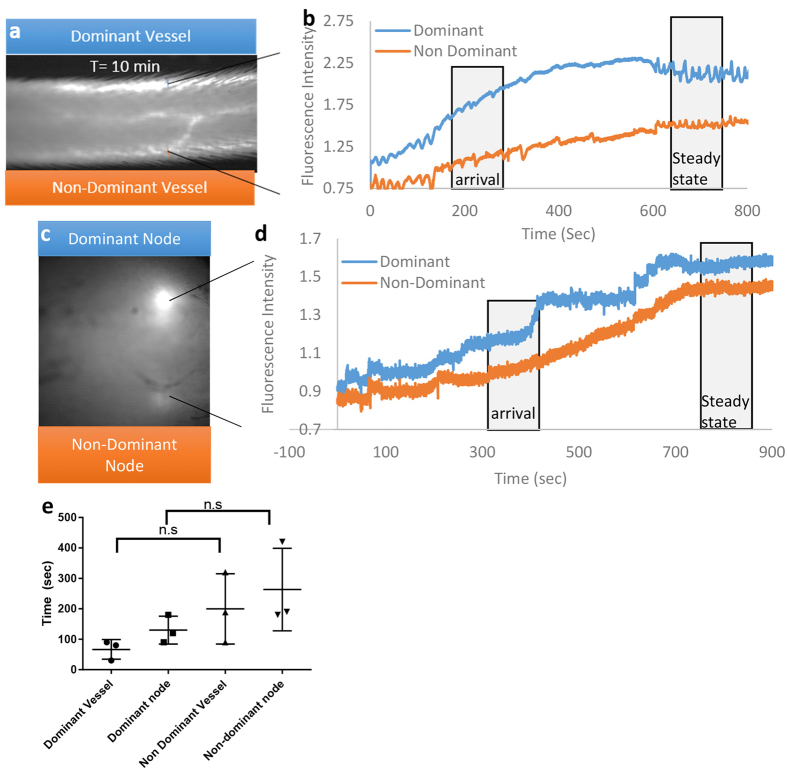
Characterization of exosomes transport *in vivo.* (**a**) Steady state fluorescence in the lymphatic collecting vessel (**b**) Intensity profile of a specified region of interest of exosome transport in a representative vessel over a 10 minute period, (**c**) Steady state fluorescence in the draining lymph node, (**d**) Intensity profile of a specified region of interest of exosome transport in a representative lymph node over a 10 minute period, (**e**) Arrival time of detectable levels of fluorescence for dominant and non-dominant collecting vessels and draining lymph nodes.

The fluorescence arrival in the dominant and non-dominant collecting vessels were analyzed and quantified from the time of injecting the exosome bolus until steady state fluorescence was achieved. The dominant vessel always had significantly higher fluorescence in all trials as compared to the non- dominant vessel (Fig. 4a; p- value <0.05) and representative line intensity profiles are shown for both vessels (Fig. 4b). The fluorescence arrival in the draining lymph nodes were analyzed and similarly, the dominant node (drained by the dominant collecting vessel) was visualized first and was brighter than the non-dominant node, which was visualized later and was fainter (Fig. 4c). Representative line intensity profiles are shown for both the draining lymph nodes (Fig. 4d). There are two distinct regions in the line intensity graphs that corresponding to a) “arrival” where there is a rapid increase in exosome transport and b) “steady state” where the exosomal transport is stable. The packet frequency in the dominant vessel was significantly higher (p-value < 0.05) at arrival as compared to the steady state while the difference in non-dominant packet frequency was not significant (Supp. Fig. 3a, p-value = 0.068). The packet frequency in the lymph nodes followed a similar pattern with a significantly higher frequency in the dominant node as compared to the non-dominant node at both the arrival and steady states (p-value <0.05, Supp. Fig. 3b). The transport times of the dominant vessel was significantly lower with fluorescence first appearing in the dominant vessel at least 30 seconds ahead of the non-dominant vessels [p-value <0.05], and this trend was replicated in the lymph nodes with fluorescence in the dominant node appearing about a 1.5 min before the non-dominant node (Fig. 4e).

To verify that the transport characteristics seen with HEY exosomes were features of lymphatic transport rather than specific to the cellular source, we injected exosomes derived from mouse lymphatic endothelial cell line (SV-LEC). We were able to recapitulate the collecting vessel and lymph node transport kinetics and characteristics (Supp. 4a–d). The arrival time in the collecting vessels and draining lymph nodes was comparable (Supp. 4e) and the packet frequency is comparable between HEY and SV-LEC exosomes.

### Characterization of exosome retention *in vivo*

**Figure 5:**
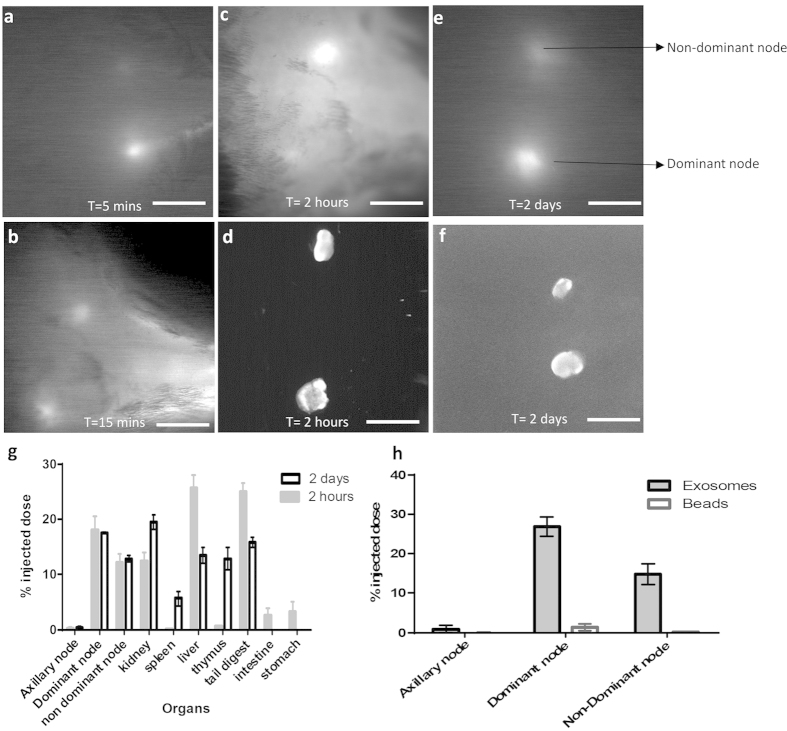
Characterization of exosome retention *in vivo.* Exosomes are detected in the node rapidly: (**a**) Only the dominant node is visible at 5 mins *in vivo*, (**b**) Both nodes are visible at 15 mins *in vivo*, (**c**) Draining lymph nodes visualized at 2 h pre-excision (in animal), (**d**) Excised lymph nodes at 2 h post injection, (**e**) Draining lymph nodes visualized at 2d pre-excision (in animal) (**f**) Excised lymph nodes at 2 days post injection, Scale = 5 mm. (**g**) Biodistribution of exosomes in mice organs analyzed at 2 hours and 2 days post injection and (**h**) quantitation of exosomes and beads retained in the lymph node 1 hour post injection as determined by fluorescence.

The exosome bolus was rapidly seen in the sciatic lymph nodes drained by the collecting lymphatics with the fluorescence arriving in both the dominant (Fig. 5a) and non-dominant node within 5 min (Fig. 5b, Supp. Video 2). The nodes reached a steady state of fluorescence much like the collecting vessels by 30 min post injection; however unlike the vessels where the fluorescence disappeared within 24 hours, the lymph node fluorescence was detectable through the skin for at least 2 days after injection (Fig. 5c,e respectively). The nodes upon excision at 2 hours and 2 days were strongly fluorescent (Fig. 5d,f respectively).

Several organs including the heart, lungs, kidney, spleen, liver, and pancreas were harvested from both mice at 2 hours and 2 days post injection and digested. The fluorescence was measured in each organ to quantify exosome retention by each organ. Exosomes were predominantly found in the injection site, draining lymph nodes, kidney and liver at 2 hours post injection and accumulated in the lymph nodes, spleen, thymus and kidney at 2 days. A significant portion was still present in the injection site in the tail (Fig. 5g).

We injected beads and SV-LEC exosomes together in mice to compare lymphatic uptake characteristics and quantified the percent of the injected dose in the lymph nodes using fluorescence on a plate reader 1 hour after injection. While exosomes were retained to a similar degree, the beads were poorly retained with the dominant node contributing to only 2% of the uptake. The non-dominant node had very poor (<1%) retention of the beads (Fig. 5h).

### Characterization of exosome retention in the draining lymph node

**Figure 6:**
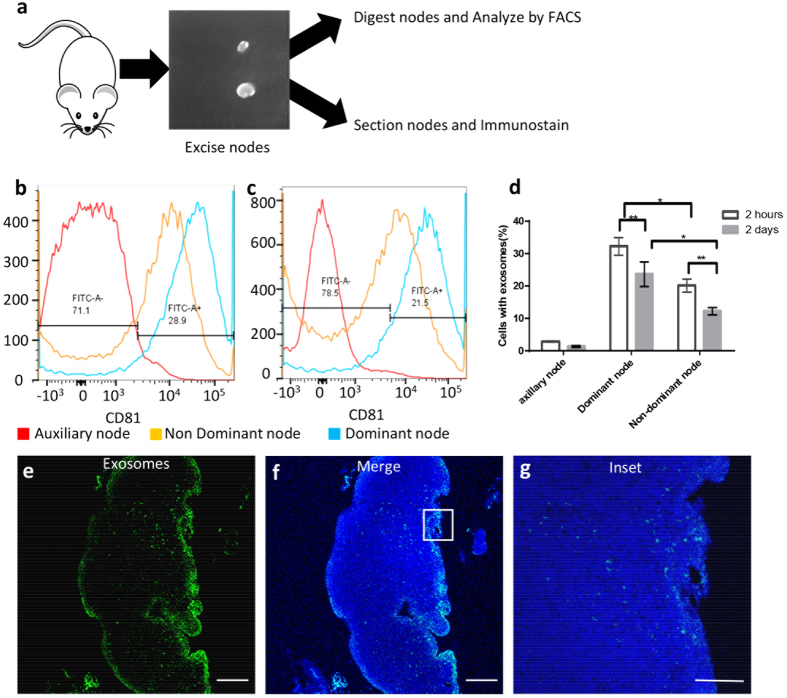
Characterization of exosome retention in the draining lymph node. (**a**) Schematic of node procession post excision from mouse (**b**) Dominant node retains a larger quantity of exosomes at 2 hours (**c**) Dominant node retains a larger quantity of exosomes at 2 days, (**d**) Quantitation of exosome retention by the dominant and non-dominant nodes at 2 hours and 2 days respectively, (**e**) Exosome localization within the node at 2 days; (**f**) Merged image with whole node nuclear staining and exosome localization, (**g**) magnified area in the node showing exosome localization. Scale bar; 10 um.

To investigate the cell populations that were responsible for uptake and retention of the exosomes in the draining lymph nodes, the dominant and non-dominant nodes were analyzed either by immunostaining or digesting the nodes and quantifying co-localization of the exosome signal with immune cells markers using FACS (Fig. 6a). The dominant lymph node contained a significantly higher proportion of exosomes than the non-dominant node, (p-value <0.05) a phenomenon that was observed at both 2 hours and 2 days (Fig. 6b,c respectively). Although the amount of exosomes retained in the cells from the digested node slightly decreased from 2 hours to 2 days post injection in both the dominant and non-dominant nodes, they still contained 10–15% of the injected exosomes and contained 1500-fold higher concentration of exosomes than the axillary lymph node which served as a control for the study as it did not directly drain the site of local exosome injection (Figs 5g and 6d). Within the draining lymph node, exosomes were predominantly present in 2 specific areas: the entire periphery of the node and in small circular areas near the periphery that corresponded to the subscapular sinus (SCS) and the follicular regions of the lymph node respectively (Fig. 6e–g).

### Role of CD11b+ and CD19+ cells in exosome uptake

**Figure 7:**
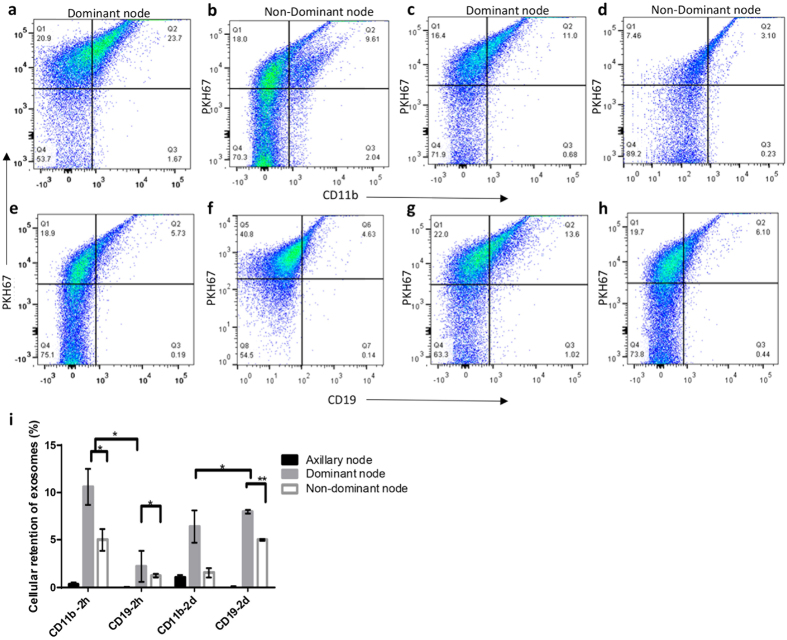
Characterization of exosome uptake by CD11b and CD19 cells in the node by flow cytometry. The dominant node was digested and stained for (**a**) CD11b at 2 h, (**c**) CD11b at 2 days, (**e**) CD19 at 2 hours, (**g**) CD19 at 2 days. The non-dominant node was stained for (**b**) CD11b at 2 h, (**d**) CD11b at 2 days, (**f**) CD19 at 2 hours, (**h**) CD19 at 2 days and (**i**) quantitation of exosome uptake by the dominant and non-dominant nodes at 2 hours and 2 days respectively expressed as a percentage of PKH67 positive cells.

To determine the primary *in vivo* targets of exosomes, we sorted the PKH positive cells and quantified the co-localization of the exosome signal with various immune cell subset markers including CD11b (Macrophages), CD19 (B-cells) CD4 (Helper T-cells), and CD8 (Killer T- cells). CD11b is abundantly expressed on the surface of monocytes and macrophages which are situated within the subcapsular sinus of the lymph node^24^. Exosomes co-localized with CD11b+ macrophages in both the dominant and non-dominant lymph nodes but the dominant node had ~2 times greater macrophage-exosome co-localization as compared to the non-dominant node at 2 hours (Fig. 7a,b). Exosome localization within macrophages was reduced by half in both the dominant and non-dominant nodes from 2 hours to 2 days (Fig. 7c,d,i).

CD19 is expressed on B-cells and is present in the B-cell follicles underlying the subcapsular sinus in the node. Exosomes were not co-localized with CD19+ B-cells at 2 hours (Fig. 7e,f) but were strongly co-localized at 2 days and the dominant node retained about ~2 times more as compared to the non-dominant node (Fig. 7g–i). There was no co-localization with either CD4+ or CD8+ T-cells at either of the time points (data not shown).

**Figure 8:**
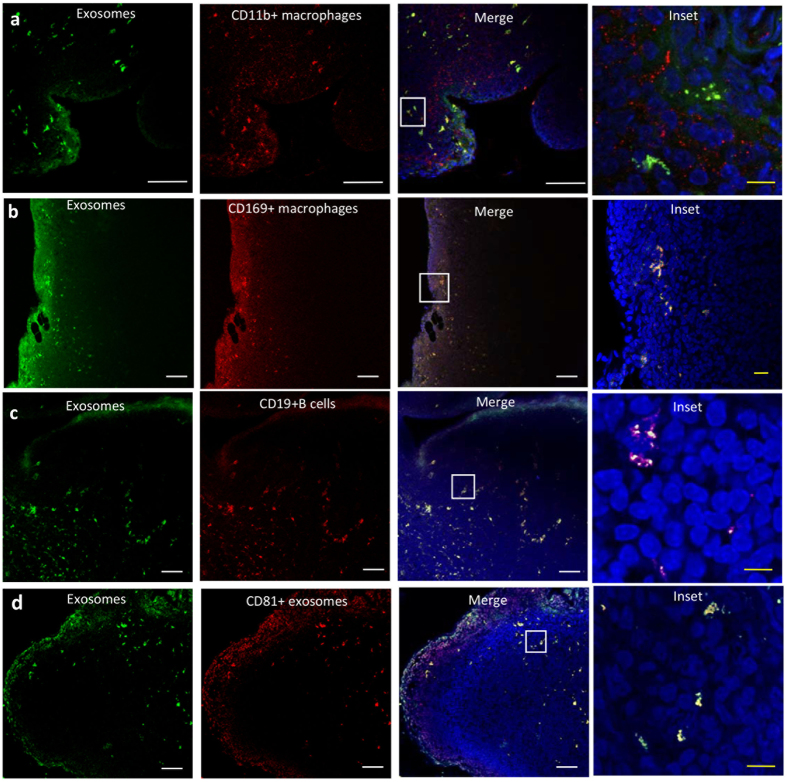
Localization of exosomes within the lymph node. Shown are serial lymph node sections at 2 days following injection of 10 ug of exosomes (green). Immune cells were identified as indicated (red) with antibodies against (**a**) CD11b (macrophages), (**b**) CD169 (macrophages), and (**c**) CD19 (B-cells), (**d**) CD81(red) was used as a secondary localization marker to confirm exosome retention in the node. White scale bar = 50 um while yellow scale bar is 5 um.

Finally, we confirmed the co-localization of exosomes with CD11b macrophages and CD19 B-cells by immunostaining frozen lymph node sections. We observed a strong co-occurrence of PKH (green) signal from the exosomes with CD11b from the macrophages and CD19 from the B-cells (Fig. 8a,c respectively). Additionally, we also checked for CD169 co-localization with exosomes to confirm macrophage mediated exosome capture (Fig. 8b). In order to ensure that the PKH signal is still present on intact exosomes, we checked for CD81 expression and found a very high degree of CD81 and PKH co-occurrence indicating that the dye was still associated with the exosomal membrane (Fig. 8d).

## Discussion

The draining lymph nodes are a stable retention site for exosomes with the quantity of exosomes retained in the nodes steadily increasing in both the dominant and non-dominant nodes from 2 hours to 2 days. Exosomes carry functional mRNA and miRNA which cause changes in gene expression in recipient cells^25^. They have also been shown to carry antigens when released from infected cells resulting in a suppression of inflammatory response *in vivo*^26^. Thus, the speed of exosome transport and retention at the node has important implications in innate immune responses. Antigen presenting cells can acquire antigens in peripheral tissues such as skin, migrate through the lymphatics to the node and activate an immune response^27^. Macrophages can release exosomes that carry specific antigens to initiate an immune response at the node: *M. tuberculosis* infected macrophages released exosomes containing mycobacterial lipoproteins which were able to stimulate a pro-inflammatory response in mice^6^. Our data suggests that exosomes can reach the lymph node and be taken up by the Cd11b+ macrophages within 5 min and thus offer a faster route for information and antigen transfer from the periphery as compared to dermal antigen presenting cell migration to the lymph node which can take hours. Additionally, this process would allow for some APCs to remain around the peripheral site of infection to survey for further signs of infection, while at the same time preemptively warning the lymph node of the danger with specific information of the nature of the infection encoded within the exosomes. This is supported by the observation that DCs continue to crawl around within the interior of the lymphatic capillary, often in directions opposite to that of lymph flow, even during inflammation^28^. Further studies elucidating the time for APC’s to package and secrete pertinent antigens and RNA molecules via exosomes are warranted to more fully understand this process. Additionally, the quantity of exosomes required to elicit a response is as yet unknown and will likely contribute the magnitude of the response developed at the node.

While both nodes exhibited stable fluorescence from the time of detection and were brightly fluorescent upon excision (at 2 hours or 2 days), a majority of exosomal uptake in the node was within the first 10 min post injection. While subtle difference in depths and diameters of the two nodes within the animal can contribute to this phenomenon, we could not observe a visible difference in the size of the nodes upon excision.

Exosomes accumulated the most in the liver, followed by the injection site and the kidney, with the stomach and intestine showing minor exosomal presence. Another study of exosomal biodistribution showed presence of exosomes in the liver and spleen 30 min post injection in the tail vein^29^. While we detected a strong signal from the liver, we were unable to detect any exosomes in the spleen until the 2 day time point. It is likely that transport via the blood and lymph will result in different biodistributions of the exosomes within whole animals and intradermal injections result in lymphatic transport with accumulations in the nodes and liver while intravenous injections result in transport by blood and accumulation in the spleen and kidneys. This is an important finding, as the *in vivo* release of exosomes from cells in the interstitium will necessarily concentrate themselves in the lymph nodes via lymphatic transport. Interestingly, in either case, a lymphoid organ is involved in exosome retention along with the liver. This phenomenon is corroborated by Saunderson *et al.*^14^, where intravenously injected exosomes accumulate in the spleen and subcutaneous injections lead to an accumulation in the lymph node 60 min post injection. We believe that the exosomes have not entered the circulation in sufficient quantity at 2 hours, due their lymphatic targeting and high levels of retention the node, thus explaining the appearance of exosomes in both the spleen and thymus only at the two day time point.

Lymphatic flow and the resulting immune response are known to be intimately connected^30^ and in certain cases of inflammation lymph flow itself can be modulated through the recruitment of immune cells to alter the contractility of the afferent vessels draining the node^31^. While lymphatic transport of exosomes has been implied in several papers^13^,^14^ they have focused on the downstream retention and effects of exosomes at the lymph node and the role of lymphatic drainage has been overlooked. Our results suggest that lymphatic flow can transfer exosomes from the periphery to the draining lymph nodes and that the transport capacity of the afferent vessels draining to the lymph node contributes to the distribution of exosomes between the nodes, with the dominating collecting vessel transporting a higher payload of exosomes to the dominant node even though both nodes appear to drain the same tissue space. This distribution remains consistent even for up to two days, and is the first study to our knowledge, that shows that the quantity of antigen in the node is correlated to the lymphatic flow to that node. Additionally, the lymphatic uptake kinetics are similar for both human and mouse cell line derived exosomes, which implies that lymphatic transport is a common mode for exosome transport from the periphery to the draining lymph nodes rather than a selective route that depends on the biological state of the cellular source. Future studies that characterize the lymphatic retention and biodistribution of exosomes from different cell types could help to understand the role of the cell of origin in the fate of exosomes *in vivo*.

HEY cells are an ovarian cancer cell line and the strong retention of HEY exosomes in the lymph node is comparable to the retention of melanoma exosomes in the node^32^. Given that ovarian cancer has one of the poorest outcomes^33^, spreads through the retroperitoneal lymphatics during metastasis^34^, and given the numerous reports of tumor exosomes modulating immune responses at the node^7^,^35^, understanding the lymphatic transport of these exosomes will further shed light on the role of lymphatic transport during cancer progression.

Rapid uptake of exosomes into the node also appears to be facilitated by active transport mechanisms in the initial lymphatic endothelial cells that are selective for exosomes, as the presence of LEC *in vitro* specifically enhanced the transport of exosomes across a permeable membrane, but not size-matched nanoparticles or lower molecular weight dextran. In fact exosome transport was 10 times higher than that of size-matched beads at 37 °C. Reduction of exosome transport at 4 °C by 80% further implies active cellular transport. This concurs with previous data that indicates that exosome uptake are actin dependent^18^ and active^36^. The molecular mechanisms that underlie exosome uptake are not well characterized and is a matter of debate. Several mechanisms including clathrin mediated endocytosis^37^, pinocytosis, plasma and membrane fusions and phagocytosis^38^ have been proposed without much consensus. Once a clearer understanding of uptake mechanisms is achieved, specific inhibitors can tease out the contributions of these pathways in exosome uptake by the lymphatic endothelial cells. Additional work to characterize the intracellular compartments as well as surface receptors that participate in exosomal shuttling will reveal potential transport mechanisms that enable selective uptake and transport. Rapid and active transport of other particles have recently been reported in lymphatic endothelial cells including lipoproteins, antigens, and albumin bound free-fatty acid, suggesting that active lymphatic trafficking, while selective, is not restricted solely to exosomes^39^,^40^,^41^,^42^.

*In vivo*, the initial lymphatics have unique button junctions that, when combined with anchoring filaments, enable fluid uptake and transport from the interstitium into the initial lymphatics^43^. Additionally, elevated transmural flow has been shown to alter expression of cell-cell junction proteins in LEC to increase uptake^44^. Thus, the injection of an exosome bolus would increase interstitial fluid pressure, lymphatic flow, and thus uptake of exosomes either directly or indirectly through active rearrangement of junctions to alter lymphatic permeability. It is likely that this passive drainage works in concert with active transcytosis to further enhance exosome clearance from the interstitial space.

Lymphatic endothelial cells (LECs) were recently shown to play an active role in scavenging antigens and presenting them to the cognate T-cells^45^. Collecting lymphatic permeability was also shown to enhance the sampling of lymphatic antigens by antigen presenting cells within the surrounding adipose tissue^46^. Thus, the selective transport of exosomes by LEC’s is a further demonstration of their active role in the establishment of immunity as they aid the exposure of the exosomes to immune cells.

The dominant and non-dominant nodes widely differ in exosome uptake by macrophages and B-cells. While this difference could be partially caused by the differing amounts of exosomes received by each node, it may not entirely explain the differences observed. Macrophages in the subcapsular sinus can capture and retain antigen from the lymphatics for up to 72 hours and then present them intact to B-cells^47^,^48^. Additionally, T-cells transferred exosomes to APC’s at the immunological synapse through cognate interactions^49^. A similar mechanism could be potentially responsible for exosome transfer, although further studies need to be conducted to understand the kinetics of exosome movement within the node, such as the time and location of RNA and protein release from the exosomes at the node and the modulation of the immune cell subsets by this mechanism. Further studies need to be conducted to see if these differences in co-localization could impact the immune response at each of these nodes in the presence of antigen which could reveal important information about the development of an innate immune response at the lymph node.

Collectively, our findings highlight the importance of lymphatic permeability and drainage in the transport of exosomes from the periphery to the lymph nodes. The work also sheds light on the immune cell subsets involved in exosome retention at the node which was hitherto unknown and could potentially be exploited in targeting the lymph node. The differential distribution of exosomes between the two draining nodes while unexpected has opened up new questions regarding distribution of antigens during an immune response and vaccine response suggesting subtle differences between the immune cell niche in the dominant and non-dominant nodes. The combination of rapid lymphatic delivery to the node and the functional consequences of exosomes on downstream cells could be a powerful combination in drug delivery, but will need a great deal of further work to unlock the full spectrum of possibilities.

## Methods

### Cell culture

Fetal bovine serum (Atlanta Biologicals, Lawrenceville, GA) was centrifuged for 15 hours at 120,000 g, 4 °C to remove exosomes and was used to make exosome free cell culture media. Human neonatal dermal lymphatic endothelial cells (LECs) were originally harvested as described previously^39^. LECs were expanded in flasks coated for 1 h with 50 μg/mL type I rat tail collagen (BD Biosciences, Bedford, MA) in 0.1% acetic acid and were cultured in EBM (Lonza, Walkersville, MD) supplemented with 20% exosome free FBS, 1% penicillin-streptomycin-amphotericin, 1% Glutamax (both from Life Technologies, Grand Island, NY), 25 mg/mL cyclic-AMP, and 1 mg/mL hydrocortisone acetate (both from Sigma, St. Louis, MO). Media was changed every 2–3 days and LECs were used for experiments at passages 9 and 10. Human ovarian adenocarcinoma cell line, HEY cells (Cedarlane Labs, Ontario, Canada) were cultured in RPMI 1640 (Mediatech, Manassas, VA) supplemented with 10% exosome free fetal bovine serum, 2 mM L-glutamine, 10 mM HEPES buffer (both from Mediatech), penicillin (100 U/ml), and streptomycin (100 μg/mL) (Life Technologies) for 48 hours and the culture media was used for isolation of exosomes by ultracentrifugation. SV-LECs were cultured as previously described^30^ and were used as a source of mouse exosomes.

### Exosome isolation and characterization

Conditioned media was collected from HEY cells at 90% confluence for exosome isolation. Briefly, the culture media was spun at 300 g, for 10 min to remove dead cells followed by a spin at 16,500 g for 20 min. The supernatant was then filtered through 0.22 μm filters and centrifuged at 120,000 g for 120 min. The pellet containing exosomes was re-suspended in a suitable volume of PBS.

The size homogeneity of vesicles obtained was checked using a Zetasizer Nano ZS90 (Malvern Instruments Ltd, Worcestershire, UK) and quantified using Pierce BCA Protein assay kit (Thermo Fisher Scientific, Waltham, MA). To analyze the expression of exosomal surface markers, 4 μm aldehyde/sulfate latex beads (Life technologies) were coated with Anti-CD9 antibody (BD Biosciences, San Diego, CA) overnight and incubated with 30 ug of exosomes. The beads were coated with biotin and the streptavidin-coated fluorescent beads were captured and assessed for surface marker expression. The exosome-bead complexes were probed with Anti Human CD81-PE or Anti human CD63-PE (BD Biosciences, San Diego, CA) and data was acquired on a BD LSR II Flow cytometer. Data analysis was performed using the FloJo software (FlowJo version 10, Ashland, OR).

### Scanning electron microscopy

Exosomes were fixed with 3.7% glutaraldehyde (Sigma–Aldrich GmbH, Taufkirchen, Germany) on carbon stubs for 15 min. After washing twice with PBS, the fixed exosomes were dehydrated with an ascending sequence of ethanol (40%, 60%, 80%, and 98%). After evaporation of ethanol, the samples were left to dry at room temperature for 24 h on a glass substrate and then analyzed by Hitachi Cold Field Emission SEM SU8200 (Hitachi High-Tec, Tokyo, Japan).

### Fluorescent labeling of exosomes

Exosomes were labeled using PKH67 Green Fluorescent Cell Linker Kit for General Cell Membrane Labeling (Sigma-Aldrich) as per the manufacturer’s instructions. Briefly, exosomes in PBS were added to 500 μL of Diluent C and 2 μL of PKH67 dye was added to 500 μL of Diluent C. The two solutions were mixed and incubated for 5 min at room temperature. 1 ml of 1% BSA was added to stop the reaction. The labeled exosomes were centrifuged at 120,000 g for 70 min and washed twice with PBS to remove excess dye.

PKH labeled exosomes were labeled with the near-infrared dye using IRDye® 800CW Protein labeling kit (Licor, Lincoln, NE) according to the manufacturer’s instructions. Briefly, exosomes in PBS were mixed with the IRDye® 800 CW NHS ester overnight and free dye was removed using Zeba desalting spin columns (Pierce).

### Transport assay and data analysis

Transwell® permeable membrane supports with 3 μm pores (Corning Life Sciences, Corning, NY) were coated for 1 h with 100 μg/mL type I rat tail collagen in PBS and LECs were seeded at a density of 100,000 cells/cm^2^ and cultured for 48 h (Fig. 2a). A fraction of the transwells were not seeded with cells and were used to determine membrane permeability (*P*_*eff_cell-free*_). Prior to transport experiments, cells were incubated for 1 h in serum-free, phenol red-free EBM (Lonza). The basal side of the monolayer was incubated with a fluorescent mix containing 20 μg/mL PKH labeled exosomes, 20 μg/mL FluoSpheres® Carboxylate-Modified 40nm Microspheres, 5 μg/mL 3 kDa Cascade Blue dextran (both from Life Technologies) for 1 h. Samples containing transported fluorescent exosomes and beads were collected from the apical side. In a subset of experiments, transport time varied from 5 to 30 min instead of 1 h. Fluorescence was measured using a Synergy™ H4 Multi-Mode Plate Reader (Biotek, Winooski, VT) and was used to calculate relative concentration based on a standard curve generated from the fluorescent mix. The effective permeability of exosomes, beads and dextran were calculated using the following equation:

*P*_*eff*_ = *Js*Δ*C*⋅*S*, where *Js* is the flux, ∆ *C* is the concentration gradient, and *S* is the surface area^39^. After samples were removed from the apical side of the transwell for fluorescence measurement and the calculation of *P*_*eff*_, membranes containing LECs were rinsed twice with PBS, fixed with 4% PFA, stained with DAPI and Alexa Fluor® 647 Phalloidin (Thermo Fisher Scientific) and mounted on glass slides for imaging on a Zeiss LSM 700 (Zeiss, Thornwood, NY). The fluorescence in the images was quantified using the Image J analysis software (v 1.4.1, NIH).

### Optimization of Near-infrared (NIR) imaging of exosomes

In order to characterize the parameters of exosome imaging in the dermis using NIR, a tissue phantom was created as described previously (Supp Fig. 2a)^15^. Additionally a node phantom was also created with two fixed depth settings; 5 mm and 7 mm to characterize the imaging setup for exosome detection at the node. The node phantom was molded in a standard petri dish using a mixture of 97.52% silicone elastomer base (Sylgard 184, Dow Corning), 2.22% Aluminum Oxide (Sigma Aldrich), and 0.26% cosmetic powder (Max Factor Crème Puff Deep Beige 42) according to previously published methods^31^.

### Near-Infrared imaging of mice

The NIR imaging system was set-up as described previously^15^. The camera was connected to a computer where the videos were acquired and analyzed by a custom LabView VI (National instruments).

Lymphatic transport of exosomes was quantified *in vivo* in the tail of eight-week-old male Balb/C mice (Charles River Laboratories, Wilmington, MA) according to procedures approved by the Georgia Institute of Technology IACUC Review Board. All methods were carried out in accordance with these approved guidelines. To minimize light scattering, a depilatory lotion was used to remove hair in the region of interest on the tail and back 1 day prior to experimentation.

The mice (n = 10) were anesthetized with Isofluorane continuously delivered through a nose cone and intradermally injected with 10 ug (10 ul of 1 ug/ul) of exosomes labeled with both PKH67 and IRDye 800CW in PBS. Care was taken to position the injection as close to the midline of the tail as possible to avoid favoring one collecting vessel over the other. The small volume of fluid injection and the use of NIR to enhance tissue penetration ensures that only fluorescence in the deeper collecting lymphatics is visible downstream of the injection site. Image acquisition began just prior to intradermal injection of the dye and the animals were imaged continuously for 20 min post-injection with a frame rate of 1 fps with a camera exposure time of 50 ms. Draining vessels, the injection site and the draining lymph nodes were imaged regularly to monitor movement of exosomes from the periphery to the nodes.

To evaluate lymphatic function in each of the mice, two parameters were measured as previously described: transport time and the average velocity of the packets traveling through the field of view of the recording site^22^. An example of fluorescence arrival in the collecting vessel can be seen in Video 1, and a plot of fluorescence intensity over time during fluorescence arrival can be seen in Fig. 4. The number of packets was measured using the plots of fluorescence intensity over time generated from two regions of interest (ROIs) in a collecting vessel and was termed the packet frequency.

### *Ex vivo* node analysis

Mice were euthanized in 2 groups; group1 (n = 5) was monitored and imaged for 2 hours post injection before euthanasia and group 2 (n = 5) was imaged for 2 hours post injection and again on days 1 and 2 before being euthanized. The draining (sacral) lymph nodes, control (axillary) lymph nodes were harvested from both the groups of mice after euthanasia. Additionally the liver, spleen, pancreas, kidney, heart, lungs, stomach, intestines, thymus and injection site were excised from one mouse in each group and was homogenized using 1.4 mm Zirconium Beads Pre-Filled Tubes (OPS Diagnostics, Lebanon, NJ) in a FastPrep 24 homogenizer (MP Biomedicals, Santa Ana, California). The supernatant was used to measure fluorescence in a Synergy™ H4 Multi-Mode Plate Reader (Biotek) to calculate exosome retention by each organ. One set of lymph nodes (both sacral and axillary) from both groups were snap-frozen in Tissue-Tek OCT (VWR, Radnor, PA) and sectioned at the Winship Cancer Institute’s Pathology Core.

### Fluorescence Confocal microscopy of lymph node sections

Frozen sections of excised sacral and axillary nodes were blocked in 10% BSA in PBS and incubated with primary antibody overnight, followed by secondary antibody for 2 h. Primary antibodies were Anti-mouse CD19, Anti-mouse CD4, Anti-mouse CD8A (all from Life Tech), Anti mouse CD14 (Sigma), Anti-mouse CD169 (Thermo Fisher Scientific), Anti-Human CD81 (BD Biosciences). These sections were detected using secondary antibodies conjugated with Alexa Fluor 647 or Alexa 680 (Life Tech) and imaged by confocal microscopy using a Zeiss LSM 700.

### Flow cytometry of nodes

Harvested lymph nodes from both group 1 (n = 3) and group 2 (n = 3) were digested with collagenase D (Roche Ltd., Mannhein, Germany) and homogenized using 70 μm pore size strainers. Cell pellets were washed staining buffer with BSA (BD Pharmingen, San Jose, CA) and centrifuged at 300 g for 1 min. To quantify exosome retention in the whole node, cells were analyzed for PKH67 positive populations using a LSR II flow cytometer (BD Biosciences, San Jose, CA). To identify cellular subsets responsible for exosome uptake, PKH67 positive cells were sorted on a FACS Aria II cytometer (BD Biosciences) and stained with monoclonal antibodies against mouse CD14, Anti-mouse CD19 and Anti-Human CD81 conjugated with, PE or AF647 for 30 min at 4 °C in the dark. Data was acquired in a LSR II flow cytometer (BD Biosciences) with compensation using single-stained cells. Data analysis was performed using FlowJo software (version 10).

### Statistical analysis

T-tests were used to compare the expression levels of tetraspanin markers between exosomes and beads (unpaired), exosome and bead transport at 37 °C and 4 °C (paired), arrival times for dominant and non-dominant vessels and nodes (paired). Exosome retention in the node and uptake by macrophages and B-cells was analyzed using paired t-tests. All analyses were run in Prism 6 (GraphPad Software Inc, La Jolla, CA) and significance was defined as p > 0.05 (not significant - ns) p ≤ 0.05 (*), p ≤ 0.01 (**), p ≤ 0.001 (***), and p ≤ 0.0001 (****). All data is presented as mean ± standard deviation.

## Additional Information

**How to cite this article**: Srinivasan, S. *et al.* Lymphatic transport of exosomes as a rapid route of information dissemination to the lymph node. *Sci. Rep.* **6**, 24436; doi: 10.1038/srep24436 (2016).

## Supplementary Material

Supplementary Movie 1

Supplementary Movie 2

Supplementary Information
