# Supplementary material for: Lymphatic transport of exosomes as a rapid route of information dissemination to the lymph node
Source: Sci Rep. 2016 Apr 18;6:24436. doi: 10.1038/srep24436 (PMC4834495; doi:10.1038/srep24436)
Supplement: Supplementary Information [file srep24436-s3.pdf]

Lymphatic transport of exosome as a rapid route of information dissemination to the lymph node

Swetha Srinivasan[1,2], Fredrik O Vannberg[1,2*], and J. Brandon Dixon[2,3]*

1. School of Biology, Georgia Institute of Technology, Atlanta, GA, USA

2. Parker H. Petit Institute for Bioengineering and Bioscience Georgia Institute of Technology, Atlanta, GA, USA

3. George W. Woodruff School of Mechanical Engineering, Georgia Institute of Technology, Atlanta, GA, USA

Correspondence:

Dr. J. Brandon Dixon

IBB 2312

315 Ferst Dr.

Atlanta, GA 30332-0405

Phone: (404) 385-3915

Fax: (404) 385-1397

dixon@gatech.edu

Keywords: exosomes, Lymphatic transport, in vivo imaging, lymph node, whole animal imaging

Word Count: 8960

Figure Count: 8

***** These authors contributed equally to this work

Supplementary Information

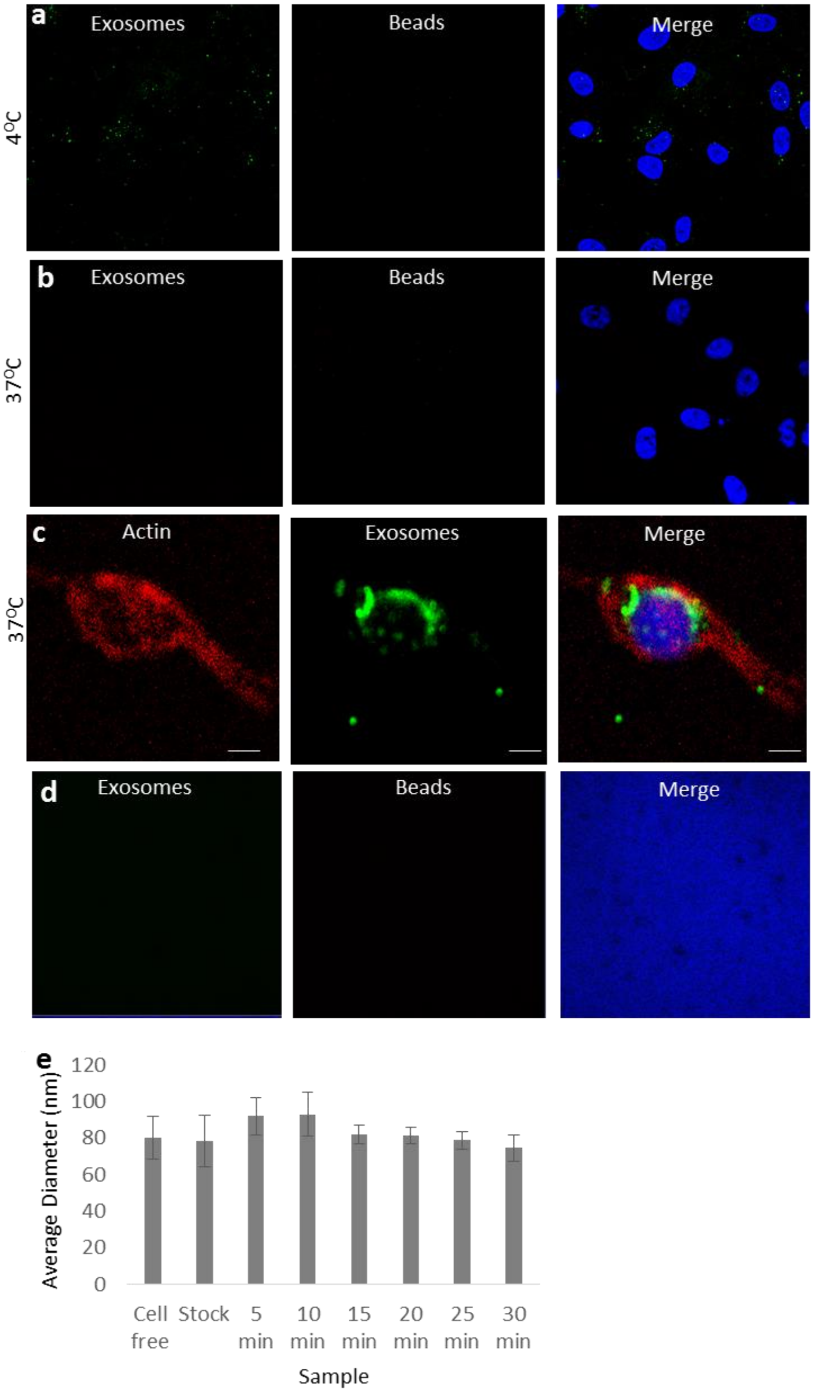

Supplementary Fig. 1: transport of exosomes and beads across the lymphatic endothelium *in vitro*
Confocal images showing a: exosomes and beads are not taken up the lymphatic endothelial cells at 4°C, b: exosomes but not beads are taken up at 37°C; c: intracellular localization of exosomes with actin; and d: the membrane does not bind to either exosomes or beads. e: Average diameter of exosome samples collected from the apical side at various time points during transport

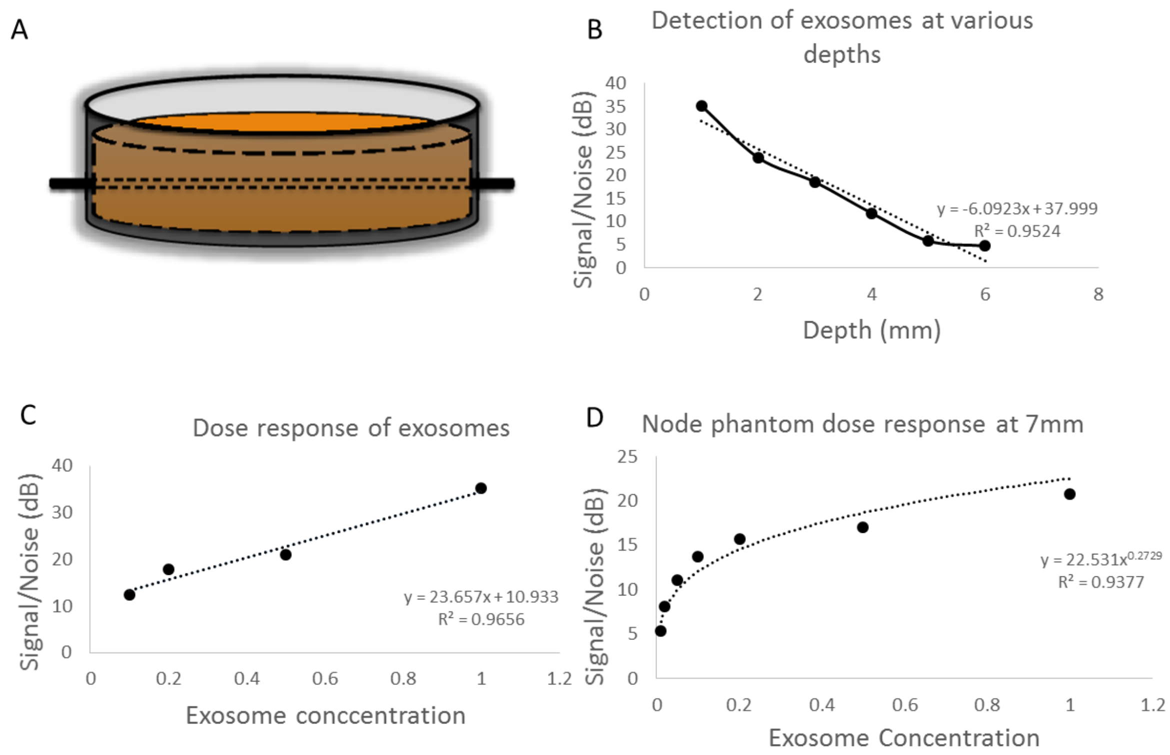

Supplementary Fig 2: Characterization of System sensitivity of labeled exosome detection. A: Description of system setup (node and tissue phantoms), B: SNR in Tissue phantom at various depths, C: Dose response of exosomes (different concentrations at 2mm depth), D: Node phantom dose response to show limit of detection at node

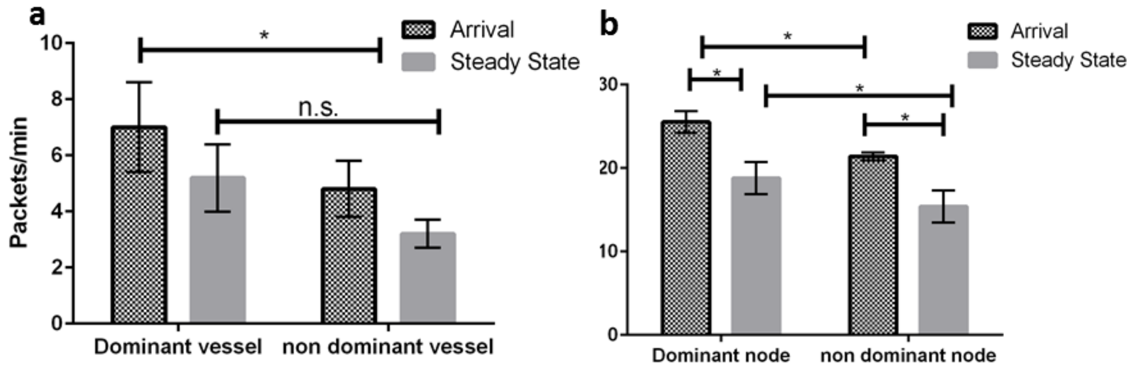

Supp Fig 3: Packet frequency at collecting vessels and draining lymph node. Packet frequencies were calculated based on number of packets detected per minute from the line diagram at A) the collecting vessels and B) the draining lymph node

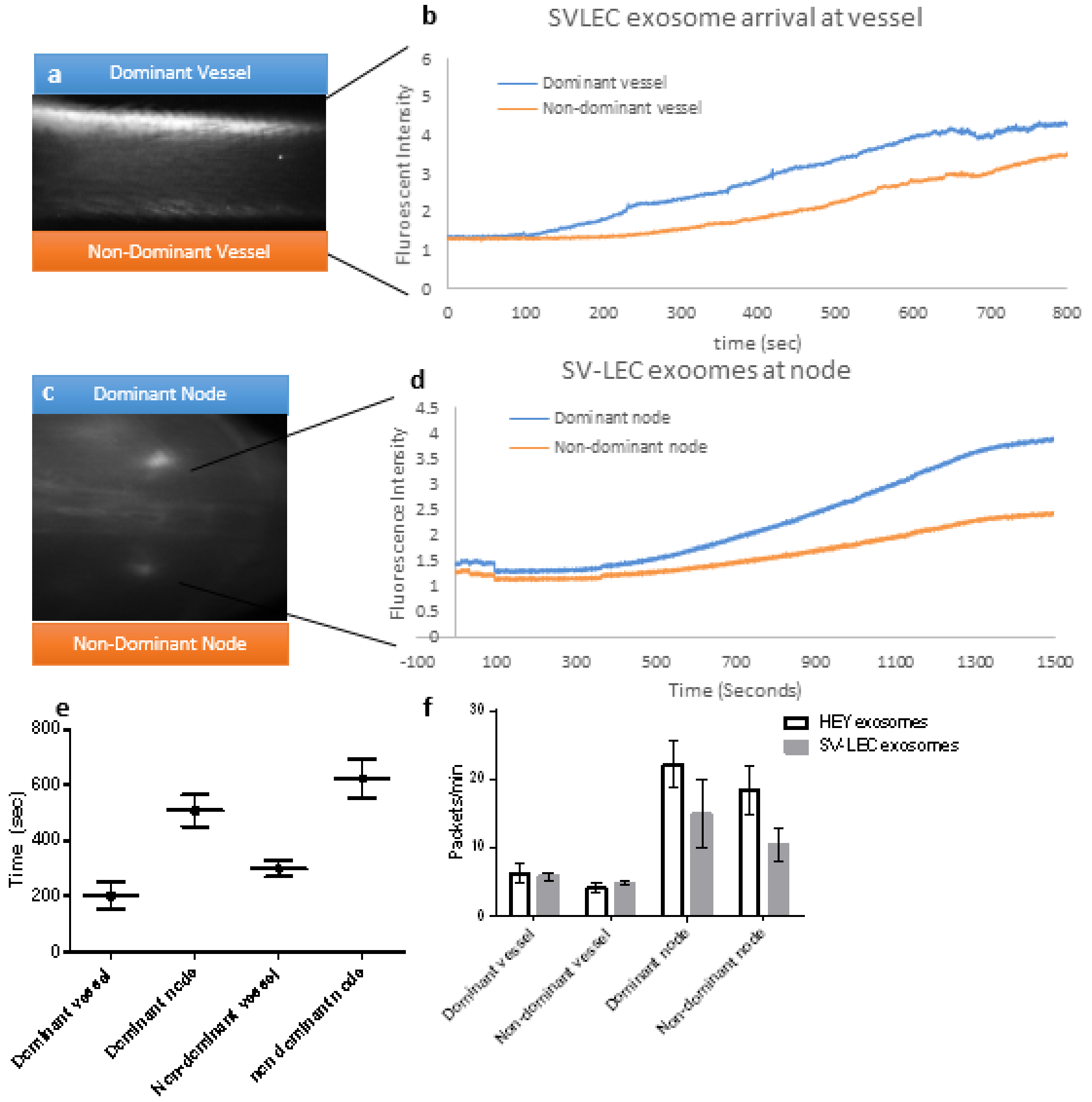

Supp. Figure 4:Characterizing SV-LEC exosome transport *in vivo* a) Steady state fluorescence in the lymphatic collecting vessel b) Intensity profile of a specified region of interest of exosome transport in a representative vessel over a 10 minute period, c) Steady state fluorescence in the draining lymph node, d) Intensity profile of a specified region of interest of exosome transport in a representative lymph node over a 10 minute period, e) Arrival time of detectable levels of fluorescence for dominant and non-dominant collecting vessels and draining lymph nodes. F) Packet frequency of HEY exosomes and SV-LEC exosomes at the collecting vessels and lymph nodes

Video legends

Supplemental Video 1: Example video of exosome arrival in the collecting vessels of a mouse 10 cm downstream from the site of intradermal injection. The dominant vessel is seen below and the non-dominant vessel is seen above. Video is played at 10X speed

Supplemental Video 2: Example video of exosome arrival in the draining (sciatic) lymph nodes of a mouse within minutes of intradermal exosome injection at the tip of the tail. The dominant node is seen above and the non-dominant vessel is seen below. Video is played at 10X speed
